# Poly[bis­(1*H*-imidazole)(μ_3_-7-oxabicyclo­[2.2.1]heptane-2,3-dicarboxyl­ato)cadmium(II)]

**DOI:** 10.1107/S1600536809021801

**Published:** 2009-06-17

**Authors:** Na Wang, Yan-Jun Wang, Qiu-Yue Lin

**Affiliations:** a Zhejiang Key Laboratory for Reactive Chemistry on Solid Surfaces, Institute of Physical Chemistry, Zhejiang Normal University, Jinhua, Zhejiang 321004, People’s Republic of China, and, College of Chemistry and Life Science, Zhejiang Normal University, Jinhua 321004, Zhejiang, People’s Republic of China

## Abstract

The title compound, [Cd(C_8_H_8_O_5_)(C_3_H_4_N_2_)_2_]_*n*_, was synthesized by the reaction of 7-oxabicyclo­[2.2.1]heptane-2,3-dicarboxylic anhydride, cadmium acetate and imidazole. The Cd^II^ atom is seven-coordinated in a distorted penta­gonal-bipyramidal configuration by five O atoms from carboxyl­ate groups of three 7-oxabicyclo­[2.2.1]heptane-2,3-dicarboxylate ligands and two N atoms from two imidazole ligands. The crystal structure is stabilized by N—H⋯O and C—H⋯O hydrogen-bonding and C—H⋯π inter­actions.

## Related literature

7-Oxabicyclo­[2.2.1]heptane-2,3-dicarboxylic anhydride (nor­cantharidin) is a lower toxicity anti­cancer drug, see: Shimi *et al.* (1982 ▶). For cobalt complexes of norcantharidin, see: Wang *et al.* (1988 ▶) and of imidazole, see: Furenlid *et al.* (1986 ▶); Zhu *et al.* (2003 ▶).
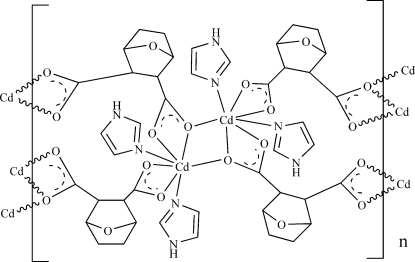


## Experimental

### 

#### Crystal data

[Cd(C_8_H_8_O_5_)(C_3_H_4_N_2_)_2_]
*M* *_r_* = 432.71
Monoclinic, 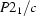

*a* = 12.5374 (16) Å
*b* = 9.6596 (13) Å
*c* = 14.1635 (17) Å
β = 112.761 (7)°
*V* = 1581.7 (3) Å^3^
*Z* = 4
Mo *K*α radiation
μ = 1.41 mm^−1^
*T* = 296 K
0.12 × 0.06 × 0.05 mm

#### Data collection

Bruker APEXII area-detector diffractometer
Absorption correction: multi-scan (*SADABS*; Sheldrick, 1996 ▶) *T*_min_ = 0.900, *T*_max_ = 0.932
10791 measured reflections
2777 independent reflections
2310 reflections with *I* > 2σ(*I*)
*R*_int_ = 0.035

#### Refinement

*R*[*F* ^2^ > 2σ(*F* ^2^)] = 0.075
*wR*(*F* ^2^) = 0.216
*S* = 1.05
2777 reflections
217 parameters
234 restraints
H-atom parameters constrained
Δρ_max_ = 2.92 e Å^−3^
Δρ_min_ = −1.26 e Å^−3^

### 

Data collection: *APEX2* (Bruker, 2006 ▶); cell refinement: *SAINT* (Bruker, 2006 ▶); data reduction: *SAINT*; program(s) used to solve structure: *SHELXS97* (Sheldrick, 2008 ▶); program(s) used to refine structure: *SHELXL97* (Sheldrick, 2008 ▶); molecular graphics: *SHELXTL* (Sheldrick, 2008 ▶); software used to prepare material for publication: *SHELXL97*.

## Supplementary Material

Crystal structure: contains datablocks I, global. DOI: 10.1107/S1600536809021801/at2804sup1.cif

Structure factors: contains datablocks I. DOI: 10.1107/S1600536809021801/at2804Isup2.hkl

Additional supplementary materials:  crystallographic information; 3D view; checkCIF report

## Figures and Tables

**Table 1:** Hydrogen-bond geometry (Å, °)

*D*—H⋯*A*	*D*—H	H⋯*A*	*D*⋯*A*	*D*—H⋯*A*
N2—H2*A*⋯O5^i^	0.86	2.09	2.830 (12)	144
N4—H4*B*⋯O1^ii^	0.86	2.44	3.012 (17)	125
N4—H4*B*⋯O2^ii^	0.86	2.05	2.818 (13)	149
C6—H6*A*⋯O4	0.98	2.56	2.92 (2)	101
C11—H11*A*⋯O5	0.93	2.34	3.239 (15)	164
C14—H14*A*⋯O2^iii^	0.93	2.55	3.358 (15)	145
C12—H12*A*⋯*Cg*5^iv^	0.93	2.76	3.565 (14)	145

Symmetry codes: (i) 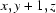; (ii) 
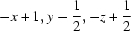
; (iii) 
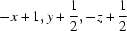
; (iv) 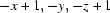. *Cg*5 is the centroid of the N3/N4/C9–C11 ring.

**Fig. 1.**
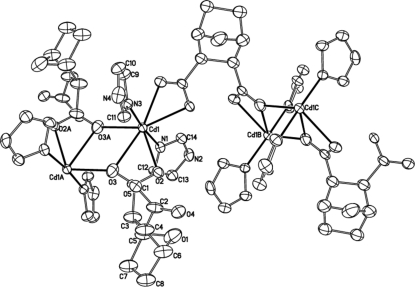
A view of the molecule of (I) showing the atom-labelling scheme with displacement ellipsoids drawn at the 30% probability.

## supplementary crystallographic information

### Comment

7-Oxabicyclo[2.2.1]heptane-2,3-dicarboxylic anhydride (norcantharidin) derived from cantharidin is a lower toxicity anticancer drug (Shimi *et al.*, 1982). Imidazole is reputed as biocatalyst and biological ligand. Several cobalt complexes of norcantharidin (Wang *et al.*, 1988) and of imidazole (Furenlid *et al.*, 1986; Zhu *et al.*, 2003) have been reported.

In the title compound, (I), (Fig. 1), the cadmium atom is seven-coordinated in a distorted pentagonal bipyramidal configuration, defined by five oxygen atoms (O2, O3, O3A, O4B, O5B) from carboxylate groups of three 7-oxabicyclo[2.2.1]heptane-2,3-dicarboxylic anhydrides and two nitrogen atoms (N1, N3) from two imidazoles. Each 7-oxabicyclo[2.2.1]heptane-2,3-dicarboxylic anhydride acts as a four-coordinared bridging linker that connects two cadmium centers.

The crystal structure is stabilized by N—H···O, C—H···O hydrogen bonding and C—H···π interactions (Table 1).

### Experimental

7-Oxabicyclo[2.2.1] heptane-2,3-dicarboxylic anhydride, cadmium acetate and imidazole were dissolved in 15 mL distilled water. The mixture was sealed in a 25 mL Teflon-lined stainless vessel and heated at 443 K for 3 d, then cooled slowly to room temperature. A crystal suitable for X-ray diffraction was obtained.

### Refinement

The H atoms bonded to C and N atoms were positioned geometrically and refined using a riding model [aromatic C—H 0.93 Å, aliphatic C—H = 0.97 (2) Å and N—H = 0.86 Å, *U*_iso_(H) = 1.2*U*_eq_(C)].

### Crystal data

**Table:**

[Cd(C_8_H_8_O_5_)(C_3_H_4_N_2_)_2_]	*F*(000) = 864
*M**_r_* = 432.71	*D*_x_ = 1.817 Mg m^−^^3^
Monoclinic, *P*2_1_/*c*	Mo *K*α radiation, λ = 0.71073 Å
Hall symbol: -P 2ybc	Cell parameters from 3164 reflections
*a* = 12.5374 (16) Å	θ = 1.8–25.0°
*b* = 9.6596 (13) Å	µ = 1.41 mm^−^^1^
*c* = 14.1635 (17) Å	*T* = 296 K
β = 112.761 (7)°	Block, colourless
*V* = 1581.7 (3) Å^3^	0.12 × 0.06 × 0.05 mm
*Z* = 4	

### Data collection

**Table:**

Bruker APEXII area-detector diffractometer	2777 independent reflections
Radiation source: fine-focus sealed tube	2310 reflections with *I* > 2σ(*I*)
graphite	*R*_int_ = 0.035
ω scans	θ_max_ = 25.0°, θ_min_ = 1.8°
Absorption correction: multi-scan (*SADABS*; Sheldrick, 1996)	*h* = −14→13
*T*_min_ = 0.900, *T*_max_ = 0.932	*k* = −9→11
10791 measured reflections	*l* = −16→16

### Refinement

**Table:**

Refinement on *F*^2^	Primary atom site location: structure-invariant direct methods
Least-squares matrix: full	Secondary atom site location: difference Fourier map
*R*[*F*^2^ > 2σ(*F*^2^)] = 0.075	Hydrogen site location: inferred from neighbouring sites
*wR*(*F*^2^) = 0.216	H-atom parameters constrained
*S* = 1.05	*w* = 1/[σ^2^(*F*_o_^2^) + (0.1141*P*)^2^ + 20.9278*P*] where *P* = (*F*_o_^2^ + 2*F*_c_^2^)/3
2777 reflections	(Δ/σ)_max_ < 0.001
217 parameters	Δρ_max_ = 2.92 e Å^−^^3^
234 restraints	Δρ_min_ = −1.26 e Å^−^^3^

### Special details

**Table:**

Geometry. All e.s.d.'s (except the e.s.d. in the dihedral angle between two l.s. planes) are estimated using the full covariance matrix. The cell e.s.d.'s are taken into account individually in the estimation of e.s.d.'s in distances, angles and torsion angles; correlations between e.s.d.'s in cell parameters are only used when they are defined by crystal symmetry. An approximate (isotropic) treatment of cell e.s.d.'s is used for estimating e.s.d.'s involving l.s. planes.
Refinement. Refinement of *F*^2^ against ALL reflections. The weighted *R*-factor *wR* and goodness of fit *S* are based on *F*^2^, conventional *R*-factors *R* are based on *F*, with *F* set to zero for negative *F*^2^. The threshold expression of *F*^2^ > σ(*F*^2^) is used only for calculating *R*-factors(gt) *etc*. and is not relevant to the choice of reflections for refinement. *R*-factors based on *F*^2^ are statistically about twice as large as those based on *F*, and *R*- factors based on ALL data will be even larger.

### Fractional atomic coordinates and isotropic or equivalent isotropic displacement parameters (Å^2^)

**Table:**

	*x*	*y*	*z*	*U*_iso_*/*U*_eq_	
Cd1	0.56124 (6)	0.12069 (7)	0.41461 (5)	0.0343 (3)	
C1	0.3497 (10)	−0.0468 (14)	0.3258 (10)	0.054 (2)	
C2	0.2845 (10)	−0.2300 (14)	0.1484 (11)	0.057 (2)	
C3	0.2272 (12)	−0.0995 (16)	0.2847 (13)	0.072 (2)	
H3A	0.2224	−0.1610	0.3380	0.086*	
C4	0.1928 (11)	−0.1894 (18)	0.1877 (12)	0.073 (2)	
H4A	0.1672	−0.2772	0.2066	0.088*	
C5	0.1415 (13)	−0.0051 (18)	0.2677 (13)	0.083 (3)	
H5A	0.1617	0.0762	0.3126	0.099*	
C6	0.0917 (13)	−0.1236 (17)	0.1263 (14)	0.080 (3)	
H6A	0.0743	−0.1363	0.0532	0.096*	
C7	0.0267 (13)	−0.0946 (18)	0.2649 (14)	0.083 (3)	
H7A	0.0478	−0.1647	0.3180	0.100*	
H7B	−0.0321	−0.0346	0.2715	0.100*	
C8	−0.0141 (13)	−0.1606 (19)	0.1553 (13)	0.081 (3)	
H8A	−0.0851	−0.1187	0.1084	0.097*	
H8B	−0.0246	−0.2599	0.1573	0.097*	
C9	0.7916 (10)	−0.0684 (14)	0.4431 (9)	0.054 (3)	
H9A	0.8443	−0.0022	0.4815	0.064*	
C10	0.8193 (11)	−0.1875 (15)	0.4113 (10)	0.060 (3)	
H10A	0.8935	−0.2187	0.4231	0.072*	
C11	0.6339 (10)	−0.1723 (13)	0.3608 (9)	0.051 (2)	
H11A	0.5560	−0.1953	0.3299	0.061*	
C12	0.3507 (10)	0.3209 (13)	0.4170 (9)	0.050 (2)	
H12A	0.3139	0.2518	0.4388	0.060*	
C13	0.3119 (11)	0.4481 (13)	0.3898 (10)	0.055 (3)	
H13A	0.2432	0.4845	0.3897	0.066*	
C14	0.4742 (10)	0.4295 (13)	0.3745 (9)	0.050 (2)	
H14A	0.5396	0.4509	0.3616	0.060*	
N1	0.4556 (8)	0.3092 (9)	0.4073 (6)	0.0389 (19)	
N2	0.3900 (9)	0.5160 (10)	0.3620 (8)	0.055 (3)	
H2A	0.3849	0.5999	0.3404	0.066*	
N3	0.6737 (7)	−0.0577 (9)	0.4109 (6)	0.0385 (19)	
N4	0.7219 (10)	−0.2529 (10)	0.3601 (8)	0.058 (3)	
H4B	0.7153	−0.3326	0.3313	0.070*	
O1	0.1232 (10)	0.0205 (13)	0.1600 (10)	0.102 (3)	
O2	0.3853 (6)	0.0273 (8)	0.2739 (6)	0.0493 (16)	
O3	0.4150 (8)	−0.0683 (11)	0.4176 (6)	0.064 (2)	
O4	0.2664 (7)	−0.2321 (9)	0.0572 (7)	0.0558 (18)	
O5	0.3769 (6)	−0.2772 (8)	0.2164 (5)	0.0425 (15)	

### Atomic displacement parameters (Å^2^)

**Table:**

	*U*^11^	*U*^22^	*U*^33^	*U*^12^	*U*^13^	*U*^23^
Cd1	0.0361 (5)	0.0323 (5)	0.0371 (5)	0.0007 (3)	0.0170 (3)	0.0026 (3)
C1	0.042 (4)	0.063 (4)	0.067 (4)	−0.001 (3)	0.032 (3)	−0.023 (4)
C2	0.039 (4)	0.064 (5)	0.076 (5)	−0.007 (4)	0.032 (4)	−0.028 (4)
C3	0.049 (4)	0.082 (5)	0.089 (5)	−0.006 (4)	0.031 (4)	−0.034 (4)
C4	0.048 (4)	0.083 (5)	0.091 (5)	−0.003 (4)	0.029 (4)	−0.035 (4)
C5	0.059 (4)	0.087 (5)	0.095 (5)	−0.004 (4)	0.023 (4)	−0.029 (4)
C6	0.054 (4)	0.085 (5)	0.094 (5)	−0.004 (4)	0.021 (4)	−0.030 (4)
C7	0.055 (5)	0.097 (5)	0.097 (5)	−0.004 (4)	0.029 (4)	−0.030 (5)
C8	0.055 (4)	0.091 (5)	0.096 (5)	−0.003 (4)	0.028 (4)	−0.032 (5)
C9	0.041 (5)	0.056 (6)	0.060 (5)	0.011 (5)	0.014 (4)	0.002 (5)
C10	0.047 (5)	0.064 (6)	0.067 (6)	0.016 (5)	0.018 (5)	0.001 (5)
C11	0.043 (4)	0.052 (5)	0.057 (5)	0.006 (4)	0.020 (4)	−0.001 (4)
C12	0.044 (5)	0.053 (5)	0.064 (5)	0.003 (4)	0.033 (4)	0.000 (4)
C13	0.051 (5)	0.052 (5)	0.068 (6)	0.007 (5)	0.029 (5)	0.003 (5)
C14	0.050 (5)	0.050 (5)	0.057 (5)	−0.002 (5)	0.029 (4)	0.001 (5)
N1	0.049 (5)	0.030 (4)	0.041 (4)	−0.001 (4)	0.021 (4)	−0.003 (4)
N2	0.071 (7)	0.032 (5)	0.065 (6)	0.014 (5)	0.031 (5)	0.013 (4)
N3	0.037 (5)	0.037 (5)	0.041 (5)	0.003 (4)	0.015 (4)	0.001 (4)
N4	0.087 (8)	0.038 (5)	0.054 (6)	0.017 (5)	0.031 (5)	−0.007 (4)
O1	0.081 (5)	0.090 (6)	0.109 (6)	0.012 (5)	0.006 (5)	0.000 (5)
O2	0.046 (3)	0.050 (4)	0.060 (4)	0.001 (3)	0.029 (3)	0.006 (3)
O3	0.066 (5)	0.086 (5)	0.046 (4)	0.018 (4)	0.028 (3)	0.004 (4)
O4	0.050 (4)	0.055 (4)	0.060 (4)	0.011 (3)	0.019 (3)	−0.002 (3)
O5	0.045 (3)	0.042 (4)	0.046 (3)	0.003 (3)	0.024 (3)	−0.005 (3)

### Geometric parameters (Å, °)

**Table:**

Cd1—N1	2.230 (9)	C7—H7A	0.9700
Cd1—N3	2.240 (9)	C7—H7B	0.9700
Cd1—O3^i^	2.333 (8)	C8—H8A	0.9700
Cd1—O5^ii^	2.476 (7)	C8—H8B	0.9700
Cd1—O4^ii^	2.487 (8)	C9—C10	1.329 (18)
Cd1—O2	2.500 (8)	C9—N3	1.372 (14)
Cd1—O3	2.599 (10)	C9—H9A	0.9300
C1—O2	1.226 (15)	C10—N4	1.316 (17)
C1—O3	1.258 (15)	C10—H10A	0.9300
C1—C3	1.506 (17)	C11—N3	1.305 (15)
C2—O4	1.222 (15)	C11—N4	1.354 (15)
C2—O5	1.271 (15)	C11—H11A	0.9300
C2—C4	1.510 (17)	C12—C13	1.323 (18)
C3—C5	1.36 (2)	C12—N1	1.379 (14)
C3—C4	1.540 (19)	C12—H12A	0.9300
C3—H3A	0.9800	C13—N2	1.357 (16)
C4—C6	1.38 (2)	C13—H13A	0.9300
C4—H4A	0.9800	C14—N1	1.306 (15)
C5—O1	1.47 (2)	C14—N2	1.304 (15)
C5—C7	1.67 (2)	C14—H14A	0.9300
C5—H5A	0.9800	N2—H2A	0.8600
C6—O1	1.476 (18)	N4—H4B	0.8600
C6—C8	1.57 (2)	O3—Cd1^i^	2.333 (8)
C6—H6A	0.9800	O4—Cd1^iii^	2.487 (8)
C7—C8	1.57 (2)	O5—Cd1^iii^	2.476 (7)
			
N1—Cd1—N3	174.2 (3)	C8—C6—H6A	112.5
N1—Cd1—O3^i^	93.7 (3)	C8—C7—C5	100.5 (13)
N3—Cd1—O3^i^	91.4 (3)	C8—C7—H7A	111.7
N1—Cd1—O5^ii^	89.6 (3)	C5—C7—H7A	111.7
N3—Cd1—O5^ii^	84.6 (3)	C8—C7—H7B	111.7
O3^i^—Cd1—O5^ii^	153.7 (3)	C5—C7—H7B	111.7
N1—Cd1—O4^ii^	90.2 (3)	H7A—C7—H7B	109.4
N3—Cd1—O4^ii^	85.8 (3)	C7—C8—C6	100.4 (12)
O3^i^—Cd1—O4^ii^	101.5 (3)	C7—C8—H8A	111.7
O5^ii^—Cd1—O4^ii^	52.3 (3)	C6—C8—H8A	111.7
N1—Cd1—O2	86.1 (3)	C7—C8—H8B	111.7
N3—Cd1—O2	94.2 (3)	C6—C8—H8B	111.7
O3^i^—Cd1—O2	117.3 (3)	H8A—C8—H8B	109.5
O5^ii^—Cd1—O2	89.0 (2)	C10—C9—N3	110.0 (12)
O4^ii^—Cd1—O2	141.1 (3)	C10—C9—H9A	125.0
N1—Cd1—O3	99.5 (3)	N3—C9—H9A	125.0
N3—Cd1—O3	85.1 (3)	N4—C10—C9	107.1 (11)
O3^i^—Cd1—O3	69.1 (4)	N4—C10—H10A	126.5
O5^ii^—Cd1—O3	136.0 (3)	C9—C10—H10A	126.5
O4^ii^—Cd1—O3	166.8 (3)	N3—C11—N4	110.6 (11)
O2—Cd1—O3	49.4 (3)	N3—C11—H11A	124.7
O2—C1—O3	118.3 (11)	N4—C11—H11A	124.7
O2—C1—C3	121.3 (13)	C13—C12—N1	107.8 (11)
O3—C1—C3	120.1 (13)	C13—C12—H12A	126.1
O4—C2—O5	122.5 (10)	N1—C12—H12A	126.1
O4—C2—C4	122.5 (13)	C12—C13—N2	107.9 (11)
O5—C2—C4	114.5 (12)	C12—C13—H13A	126.1
C5—C3—C1	117.4 (13)	N2—C13—H13A	126.1
C5—C3—C4	106.9 (13)	N1—C14—N2	111.8 (10)
C1—C3—C4	115.3 (11)	N1—C14—H14A	124.1
C5—C3—H3A	105.4	N2—C14—H14A	124.1
C1—C3—H3A	105.4	C14—N1—C12	105.7 (10)
C4—C3—H3A	105.4	C14—N1—Cd1	124.0 (7)
C6—C4—C2	122.0 (16)	C12—N1—Cd1	129.4 (8)
C6—C4—C3	100.0 (12)	C14—N2—C13	106.8 (10)
C2—C4—C3	119.0 (11)	C14—N2—H2A	126.6
C6—C4—H4A	104.6	C13—N2—H2A	126.6
C2—C4—H4A	104.6	C11—N3—C9	104.6 (10)
C3—C4—H4A	104.6	C11—N3—Cd1	123.6 (7)
C3—C5—O1	95.3 (13)	C9—N3—Cd1	131.1 (8)
C3—C5—C7	105.8 (14)	C10—N4—C11	107.7 (10)
O1—C5—C7	106.0 (12)	C10—N4—H4B	126.1
C3—C5—H5A	115.8	C11—N4—H4B	126.1
O1—C5—H5A	115.8	C6—O1—C5	95.3 (13)
C7—C5—H5A	115.8	C1—O2—Cd1	98.4 (7)
C4—C6—O1	99.4 (12)	C1—O3—Cd1^i^	149.9 (8)
C4—C6—C8	113.0 (16)	C1—O3—Cd1	92.7 (8)
O1—C6—C8	106.1 (13)	Cd1^i^—O3—Cd1	110.9 (4)
C4—C6—H6A	112.5	C2—O4—Cd1^iii^	92.6 (7)
O1—C6—H6A	112.5	C2—O5—Cd1^iii^	91.9 (6)
			
O2—C1—C3—C5	−67.4 (19)	C10—C9—N3—Cd1	−170.3 (9)
O3—C1—C3—C5	106.4 (18)	N1—Cd1—N3—C11	−105 (3)
O2—C1—C3—C4	60.1 (19)	O3^i^—Cd1—N3—C11	105.9 (9)
O3—C1—C3—C4	−126.2 (15)	O5^ii^—Cd1—N3—C11	−100.2 (9)
O4—C2—C4—C6	−15 (2)	O4^ii^—Cd1—N3—C11	−152.6 (9)
O5—C2—C4—C6	173.5 (13)	O2—Cd1—N3—C11	−11.6 (9)
O4—C2—C4—C3	−140.1 (15)	O3—Cd1—N3—C11	37.0 (9)
O5—C2—C4—C3	48 (2)	N1—Cd1—N3—C9	64 (3)
C5—C3—C4—C6	3.3 (19)	O3^i^—Cd1—N3—C9	−84.9 (10)
C1—C3—C4—C6	−129.2 (15)	O5^ii^—Cd1—N3—C9	69.0 (10)
C5—C3—C4—C2	138.9 (16)	O4^ii^—Cd1—N3—C9	16.6 (10)
C1—C3—C4—C2	6(2)	O2—Cd1—N3—C9	157.6 (10)
C1—C3—C5—O1	91.7 (15)	O3—Cd1—N3—C9	−153.8 (10)
C4—C3—C5—O1	−39.6 (16)	C9—C10—N4—C11	0.0 (15)
C1—C3—C5—C7	−160.0 (14)	N3—C11—N4—C10	0.2 (14)
C4—C3—C5—C7	68.7 (17)	C4—C6—O1—C5	−60.7 (15)
C2—C4—C6—O1	−99.0 (17)	C8—C6—O1—C5	56.7 (14)
C3—C4—C6—O1	34.8 (16)	C3—C5—O1—C6	60.4 (13)
C2—C4—C6—C8	149.0 (14)	C7—C5—O1—C6	−47.8 (13)
C3—C4—C6—C8	−77.2 (16)	O3—C1—O2—Cd1	−11.1 (12)
C3—C5—C7—C8	−76.9 (17)	C3—C1—O2—Cd1	162.8 (10)
O1—C5—C7—C8	23.5 (16)	N1—Cd1—O2—C1	−99.8 (7)
C5—C7—C8—C6	10.1 (16)	N3—Cd1—O2—C1	86.1 (7)
C4—C6—C8—C7	65.5 (17)	O3^i^—Cd1—O2—C1	−7.6 (8)
O1—C6—C8—C7	−42.4 (17)	O5^ii^—Cd1—O2—C1	170.6 (7)
N3—C9—C10—N4	−0.3 (15)	O4^ii^—Cd1—O2—C1	174.6 (7)
N1—C12—C13—N2	0.7 (14)	O3—Cd1—O2—C1	6.2 (7)
N2—C14—N1—C12	−0.4 (13)	O2—C1—O3—Cd1^i^	153.0 (13)
N2—C14—N1—Cd1	169.4 (8)	C3—C1—O3—Cd1^i^	−21 (2)
C13—C12—N1—C14	−0.2 (13)	O2—C1—O3—Cd1	10.5 (11)
C13—C12—N1—Cd1	−169.3 (8)	C3—C1—O3—Cd1	−163.4 (10)
N3—Cd1—N1—C14	−18 (3)	N1—Cd1—O3—C1	70.6 (7)
O3^i^—Cd1—N1—C14	131.6 (9)	N3—Cd1—O3—C1	−105.8 (7)
O5^ii^—Cd1—N1—C14	−22.2 (9)	O3^i^—Cd1—O3—C1	160.9 (9)
O4^ii^—Cd1—N1—C14	30.1 (9)	O5^ii^—Cd1—O3—C1	−28.7 (8)
O2—Cd1—N1—C14	−111.2 (9)	O4^ii^—Cd1—O3—C1	−152.6 (11)
O3—Cd1—N1—C14	−158.9 (9)	O2—Cd1—O3—C1	−6.0 (6)
N3—Cd1—N1—C12	150 (3)	N1—Cd1—O3—Cd1^i^	−90.3 (4)
O3^i^—Cd1—N1—C12	−61.1 (10)	N3—Cd1—O3—Cd1^i^	93.3 (4)
O5^ii^—Cd1—N1—C12	145.1 (9)	O3^i^—Cd1—O3—Cd1^i^	0.0
O4^ii^—Cd1—N1—C12	−162.7 (9)	O5^ii^—Cd1—O3—Cd1^i^	170.4 (3)
O2—Cd1—N1—C12	56.1 (9)	O4^ii^—Cd1—O3—Cd1^i^	46.5 (14)
O3—Cd1—N1—C12	8.4 (10)	O2—Cd1—O3—Cd1^i^	−166.9 (5)
N1—C14—N2—C13	0.8 (14)	O5—C2—O4—Cd1^iii^	9.0 (13)
C12—C13—N2—C14	−0.9 (14)	C4—C2—O4—Cd1^iii^	−162.4 (13)
N4—C11—N3—C9	−0.4 (13)	O4—C2—O5—Cd1^iii^	−9.0 (13)
N4—C11—N3—Cd1	171.2 (7)	C4—C2—O5—Cd1^iii^	163.0 (11)
C10—C9—N3—C11	0.4 (14)		

Symmetry codes: (i) −*x*+1, −*y*, −*z*+1; (ii) −*x*+1, *y*+1/2, −*z*+1/2; (iii) −*x*+1, *y*−1/2, −*z*+1/2.

### Hydrogen-bond geometry (Å, °)

**Table:**

*D*—H···*A*	*D*—H	H···*A*	*D*···*A*	*D*—H···*A*
N2—H2A···O5^iv^	0.86	2.09	2.830 (12)	144
N4—H4B···O1^iii^	0.86	2.44	3.012 (17)	125
N4—H4B···O2^iii^	0.86	2.05	2.818 (13)	149
C6—H6A···O4	0.98	2.56	2.92 (2)	101
C11—H11A···O5	0.93	2.34	3.239 (15)	164
C14—H14A···O2^ii^	0.93	2.55	3.358 (15)	145
C12—H12A···Cg5^i^	0.93	2.76	3.565 (14)	145

Symmetry codes: (iv) *x*, *y*+1, *z*; (iii) −*x*+1, *y*−1/2, −*z*+1/2; (ii) −*x*+1, *y*+1/2, −*z*+1/2; (i) −*x*+1, −*y*, −*z*+1.
